# Potential Usefulness of Lifetime Globotriaosylsphingosine Exposure at Diagnosis and Baseline Modified Disease Severity Score in Early-Diagnosed Patients With Fabry Disease

**DOI:** 10.7759/cureus.61380

**Published:** 2024-05-30

**Authors:** Junko Hotta, Yukiko Jogu, Haruka Bamba, Yasuhiro Izumiya, Masaharu Kudo, Takumi Imai, Hitoshi Sakuraba, Takashi Hamazaki, Toshiyuki Seto

**Affiliations:** 1 Department of Medical Genetics, Osaka Metropolitan University Graduate School of Medicine, Osaka, JPN; 2 Department of Pediatrics, Osaka Metropolitan University Graduate School of Medicine, Osaka, JPN; 3 Department of Cardiovascular Medicine, Osaka Metropolitan University Graduate School of Medicine, Osaka, JPN; 4 Department of Medical Statistics, Osaka Metropolitan University Graduate School of Medicine, Osaka, JPN; 5 Department of Clinical Genetics, Meiji Pharmaceutical University, Tokyo, JPN

**Keywords:** ds3, mssi, lifetime lyso-gb3 exposure, lyso-gb3, fabry disease

## Abstract

Background: Fabry disease (FD) is a lysosomal storage disease caused by a deficit of α-galactosidase A (GAL). Recently, plasma globotriaosylsphingosine (lyso-Gb3), a pathogenic analog of a substrate of GAL, has been suggested as a potential biomarker for FD, and disease severity scores, such as the Mainz Severity Score Index (MSSI), the Disease Severity Scoring System (DS3), and FASTEX (FAbry STabilization indEX), are useful tools for evaluating the severity of signs and symptoms in symptomatic FD patients. However, a more useful method of evaluating disease severity in early-diagnosed FD patients such as children, adult females, and asymptomatic patients is needed. Here, we proposed modified MSSI and DS3 scores to which we added phenotype, urinary mulberry bodies, and history of past pain attacks and examined the clinical usefulness of lyso-Gb3 and modified scores for early-diagnosed FD patients.

Result: In 13 early-diagnosed FD patients, we developed modified MSSI and DS3 scores and examined the correlation of lifetime lyso-Gb3 exposure at diagnosis with the conventional or modified scores. Lifetime lyso-Gb3 exposure was positively correlated only with the modified DS3 score. Additionally, we examined the long-term changes in plasma lyso-Gb3 concentration and in conventional MSSI, DS3, and FASTEX. In males, plasma lyso-Gb3 concentration decreased more rapidly than in females. In all patients, the severity scores were mild and remained nearly stable throughout the follow-up period.

Conclusion: Our data suggest that lifetime lyso-Gb3 exposure and the modified DS3 score are useful in early-diagnosed patients.

## Introduction

Fabry disease (FD; OMIM #301500) is an X-linked lysosomal storage disease caused by a pathogenic mutation in the *GLA* gene encoding the lysosomal enzyme α-galactosidase A (GAL; EC 3.2.1.22) [1]. Deficiency of GAL activity leads to the accumulation of globotriaosylceramide (GL3), resulting in the development of life-threatening conditions such as progressive renal failure, chronic heart failure due to hypertrophic cardiomyopathy, and cerebral infarction. FD is classified into classical and late-onset forms in accordance with GAL activity and clinical features. Additionally, the disease types can be estimated according to the *GLA* mutation previously reported. Patients with the classical form have acroparesthesia and hypohidrosis in childhood. Furthermore, in adulthood, they have renal and cardiac complications and cerebral infarction [2-4]. In contrast, patients with the late-onset form have no symptoms in childhood and have cardiac or renal complications in the fourth to seventh decades of life [5,6]. Generally, heterozygous females have a milder phenotype than males [7,8] and have a wide spectrum of symptoms [9,10]. The clinical course and complications vary widely across patients with the same type of disease.

Enzyme replacement therapy (ERT) was first approved in the European Union in 2001 and is now available worldwide [11-13]. Treatment guidelines currently emphasize the need for ERT to be started as early as possible after onset and for it to be continued with long-term monitoring [4,14]. In this context, the availability of a biomarker would facilitate not only the early diagnosis of FD but also the long-term clinical evaluation during ERT. Recently, plasma globotriaosylsphingosine (lyso-Gb3), a deacylated derivative of GL3, has been suggested as a potentially useful biomarker of FD [15,16], and correlations between lifetime exposure to this derivative and disease severity have been studied [17]. Also, several severity scoring systems have been reported. The Mainz Severity Score Index (MSSI) and the Disease Severity Scoring System (DS3) have been developed for evaluating clinical severity, and FASTEX (FAbry STabilization indEX) has been developed for assessing clinical stability during follow-up [18-20].

Since the development of ERT, physicians’ awareness of FD has increased, and FD patients diagnosed in childhood or adolescence have increased. There is as yet no unified view on when to initiate treatment before the onset of clinical signs [21,22]. However, we believe that early diagnosis and initiation of treatment before irreversible organ damage occurs are imperative because histological changes begin in the fetal period [23]. Therefore, a modified scoring system is required to appropriately evaluate the clinical features of early-diagnosed patients.

Here, to gain a better understanding of the clinical usefulness of lyso-Gb3 as a biomarker of FD and to explore the development of a modified severity score that could be used for the more sensitive assessment of early-diagnosed FD patients, we examined the correlation between disease severity score and lifetime lyso-Gb3 exposure at diagnosis. We also examined the long-term changes in plasma lyso-Gb3 concentration in response to treatment with ERT or chemical chaperone therapy.

An earlier version of this article was previously posted to the Research Square preprint server on April 13, 2023.

## Materials and methods

Subjects

This was a single-center, retrospective analysis of 13 consecutive treatment-naïve patients with FD who received periodic pediatric, cardiologic, or nephrologic checkups under the supervision of certified medical geneticists and specialists at the Department of Pediatrics, Osaka Metropolitan University (Osaka, Japan) from August 2016 to March 2021. The 13 patients were from a total of five families. Four of the patients were males (age, nine to 20 years; median age, 14.0 years) and nine were females (age, seven to 51 years, median age, 44.0 years). Of the patients, pediatric patients were five. The patients were followed up every three to six months for the first year, then every six to 12 months thereafter at Osaka Metropolitan University Hospital (Osaka, Japan), and the average follow-up period for the whole cohort was 3.3 years. Each patient was suspected of having FD on the basis of semiology, family history, the existence of at-risk relatives, detection of urinary mulberry bodies, and plasma lyso-Gb3 measurements. The patients were diagnosed by GAL activity testing and genetic testing. Patients were included in the study if they had a genetically confirmed diagnosis of FD and their clinical record included their plasma lyso-Gb3 concentration at diagnosis; patients were excluded if their clinical record excluded their plasma lyso-Gb3 concentration at diagnosis.

The Institutional Review Board of Osaka Metropolitan University Hospital approved this study, and the tenets of the Declaration of Helsinki were followed. Written informed consent for the processing of personal data was obtained from each patient or the parents of patients under the age of 18 years.

Determination of plasma lyso-Gb3 concentration

Blood samples were collected from the patients at diagnostic or follow-up blood examinations in the outpatient clinic of Osaka Metropolitan University Hospital. After collection, the samples were centrifuged for 10 minutes (4°C, 2150 g); plasma and serum were then collected separately and stored at −80°C until analysis. Plasma lyso-Gb3 concentration was measured by liquid chromatography-electrospray ionization-tandem mass spectrometry (LC-ESI-MS/MS) in multiple reaction monitoring modes. The normal range was determined to be 0.14-0.75 nmol/L.

Calculation of lifetime lyso-Gb3 exposure at diagnosis

For each patient, a longitudinal analysis of plasma lyso-Gb3 concentration was conducted from diagnosis (day 0, baseline) to the latest follow-up.

Additionally, each patient’s lifetime exposure to lyso-Gb3 (U) was calculated by multiplying their age at diagnosis in years by their baseline lyso-Gb3 concentration in accordance with the method used in a previous report [17].

Genotyping of GLA and measurement of GAL activity

Genomic DNA was purified from leukocytes. Seven exons of the *GLA* gene were amplified by polymerase chain reaction (PCR) using the appropriate primers [24,25]. PCR and direct sequencing were performed as previously described [25]. GAL activity was measured in peripheral blood leukocytes by using a method previously described and the normal range was determined to be 20-80 nmol/h/mg protein [26].

Qualitative evaluation of urinary mulberry bodies and proteinuria

Urine samples were collected from the patients at diagnostic or follow-up urine examinations in the outpatient clinic of Osaka Metropolitan University Hospital. The urine sample (10 mL) was centrifuged and the supernatant was removed to obtain 200 μL of concentrated urine sample; 15 μL of the sample was used to identify urinary mulberry bodies (uMBs). By using an ECLIPSE Ci Series microscope (Nikon, Tokyo, Japan), we established a simple qualitative evaluation system for uMBs that used three grades depending on the number of uMBs detected per whole field at a magnification of 200, namely, grade 1 (one to nine mulberry bodies), grade 2 (10 to 99 mulberry bodies), and grade 3 (≥100 mulberry bodies). Urinary protein was measured by dipstick test.

Evaluation of disease severity and stability

To evaluate clinical severity and stability, three scoring systems were used: the MSSI, the DS3, and the FASTEX. The MSSI was developed to assess the severity of the signs and symptoms of FD patients and consists of four components (general, neurological, renal, and cardiovascular) [18]. The DS3 was developed for easy use in general FD patients. It consists of five domains: four clinical domains (peripheral nervous system, central nervous system, renal, and cardiac) and a patient-reported domain [19]. The FASTEX was developed to assess the clinical stability or disease progression between two consecutive evaluations. It consists of three domains (nervous, renal, and cardiac). A score change of >20% between two visits is considered to indicate disease instability [20].

To investigate whether the modified severity scores were potentially more useful in early-diagnosed FD patients, we added one point each for a history of past pain attacks, the phenotype of classical form, which was determined based on clinical feature, genotype, or family history, as well as points for the number of uMBs (grade 1, one point; grade 2, two points; grade 3, three points), to the conventional DS3 and MSSI scores.

Statistical analysis

Participants’ demographics were described as percentages (counts) in the case of categorical variables and as median (range) for continuous variables. They were compared between males and females using Fisher’s exact test or the Mann-Whitney U-test. Longitudinal changes in plasma lyso-Gb3 during treatment were analyzed using a linear regression model, including month variable with a restricted cubic spline function. Values of plasma lyso-Gb3 were modeled on a logarithmic scale. Longitudinal changes in conventional MSSI and DS3 scores during treatment were plotted against follow-up months. The correlation between lifetime lyso-Gb3 exposure and disease severity scores or modified severity scores was examined using Spearman’s rank correlation. All analyses were performed with R version 4.0.4 (R Foundation for Statistical Computing, Vienna, Austria) and the EZR graphical user interface for R (Saitama Medical Center, Jichi Medical University). EZR is a modified version of R Commander (version 2.7-1) that provides statistical functions that are frequently used in biostatistics [27].

## Results

Patient characteristics

A total of 13 patients (four males and nine females) from five families were enrolled in the study (Table 1). In terms of genotype, all the patients except for those in family 5 (Table 2) were diagnosed with a pathogenic variant of classical FD, as confirmed by referencing the online mutation database Human Gene Mutation Database (HGMD) Professional 2020.4 [28]. Family 5 was diagnosed with a novel variant of late-onset (cardiac) FD after consideration of their semiology, the results of biochemical analyses, and their family history (e.g., patient 9’s father had been diagnosed with FD based on a GAL enzyme analysis and deposits in cardiomyocytes).

**Table 1:** Baseline characteristics. Categorical variables are represented as percentages (counts) and continuous variables are represented as medians (ranges). Abbreviations: DS3, Disease Severity Scoring System; GAL, α-galactosidase A; lyso-Gb3, globotriaosylsphingosine; MSSI, Mainz Severity Score Index.

	Males (n = 4)	Females (n = 9)	P-value
Classical form, % (N)	75.0 (3/4)	55.6 (5/9)	1.00
Age, years	14.0 (9–20)	44.0 (7–51)	0.10
GAL, nmol h^–1^ mg protein^–1^	0.55 (0.0–6.0)	29.0 (3.5–40.0)	0.01
Lyso-Gb3, nmol/L	150.5 (10.3–261.9)	5.7 (1.0–16.8)	0.01
Lifetime lyso-Gb3 exposure, U	1638.3 (205.7–3666.3)	162.6 (15.8–737.9)	0.02
Acroparesthesia, % (N)	75.0 (3/4)	33.3 (3/9)	0.27
Hypohidrosis, % (N)	75.0 (3/4)	22.2 (2/9)	0.22
Angiokeratoma, % (N)	25.0 (1/4)	0 (0/9)	0.31
Cornea verticillata, % (N)	0 (0/4)	1 (1/7)	1.00
White matter lesion, % (N)	0 (0/4)	12.5 (1/8)	1.00
Left ventricular posterior wall thickness, mm	7.0 (6.0–9.0)	7.0 (5.0–9.0)	0.92
Interventricular septum thickness, mm	7.0 (5.0–9.0)	8.0 (4.0-12.0)	0.55
Conventional DS3 score	4.5 (2.0–6.7)	1.3 (1.3–5.7)	0.03
Conventional MSSI score	5.5 (2.0–9.0)	1.0 (0.0–11.0)	0.10

**Table 2:** Clinical manifestations, laboratory data, and conventional disease severity scores in Fabry disease patients at diagnosis. ^1^ Grade 1 (one to nine mulberry bodies); grade 2 (10 to 99 mulberry bodies); grade 3 (≥100 mulberry bodies). ^2^ MSSI score; the maximum score is 76; severity is classified as mild (≤18), moderate (19-38), or severe (>38). ^3^ ​​​​DS3 score; the maximum score is 32; severity is classified as mild (<8), moderate/marked disease (8-12), or severe (>12). Abbreviations: DS3, Disease Severity Scoring System; ERT, enzyme replacement therapy; GAL, α-galactosidase A; lyso-Gb3, globotriaosylsphingosine; MRI, magnetic resonance imaging; MSSI, Mainz Severity Score Index.

Family no.	Phenotype (genotype)	Patient no.	Age at diagnosis (years)	Sex	Main symptoms	GAL (nmol h^–1^ mg protein^–1^)	Plasma lyso-Gb3 concentration (nmol/L)	Lifetime lyso-Gb3 exposure (U)	Mulberry bodies in urine (grade)^1^	Proteinuria	DS3		MSSI		Treatment
											Score^2^	Severity	Score^3^	Severity	
1	Classical (C378F)	1	39	F	History of past pain attacks, acroparesthesia	29.8	9.4	365.4	1	None	2.0	Mild	2	Mild	ERT (agalsidase beta)
		2	12	F	Pain attack, acroparesthesia, hypohidrosis	3.5	5.7	68.1	1	None	4.7	Mild	7	Mild	ERT (agalsidase beta)
		3	9	M	Pain attack, acroparesthesia, hypohidrosis, general fatigue	0.3	187.3	1685.8	1	None	6.0	Mild	7	Mild	ERT (agalsidase beta)
2	Classical (G35E)	4	46	F	No symptoms	32.1	13.6	626.4	1	None	1.3	Mild	0	Mild	Chaperone therapy (migalastat)
		5	14	M	Pain attack, acroparesthesia	0.8	113.6	1590.8	1	None	3.0	Mild	4	Mild	ERT (agalsidase beta)
3	Classical (S148N)	6	43	F	Idiopathic sudden deafness	25.0	17	731.0	2	None	1.3	Mild	0	Mild	ERT (agalsidase beta)
		7	14	M	Pain attack, hypohidrosis, orthostatic dysregulation	0.0	261.9	3666.6	2	None	6.7	Mild	9	Mild	ERT (agalsidase beta)
4	Classical (N263S)	8	22	F	Pain attack, acroparesthesia, hypohidrosis, general fatigue	32.0	7.4	162.8	2	None	5.7	Mild	10	Mild	ERT (agalsidase beta BS)
5	Late-onset (IVS3+4A>G)	9	51	F	No symptoms	13.0	3.7	188.7	1	None	1.3	Mild	1	Mild	ERT (agalsidase alfa)
		10	20	M	No symptoms (abnormality on T1 mapping cardiac MRI)	6.0	12	240.0	1	None	2.0	Mild	2	Mild	ERT (agalsidase alfa)
		11	48	F	No symptoms	29.0	2.4	112.7	1	None	1.3	Mild	0	Mild	ERT-naïve
		12	45	F	No symptoms	20.0	2.5	46.2	1	None	1.3	Mild	1	Mild	ERT-naïve
		13	7	F	No symptoms	40.0	0.91	20.3	0	None	1.3	Mild	0	Mild	ERT-naïve

All patients had no main organ symptoms such as left ventricular hypertrophy in echocardiogram, proteinuria, renal failure in urine test or blood test, or history of cerebral infarction. Thus, we regarded their condition as not an advanced state and we defined them as early-diagnosed patients. We considered treating the asymptomatic patient in response to her request, taking into account the laboratory data, early markers, family history, and age after an assessment had been provided. After diagnosis, nine patients received ERT and one patient received chemical chaperone therapy [29]. Three patients did not start treatment. The baseline conventional DS3 scores, plasma lyso-Gb3 concentration, and lifetime lyso-Gb3 exposure in males were all significantly higher than in females. uMBs were detected in all patients except the youngest girl (Table 2) and persisted throughout the follow-up period (data not shown).

Correlation between disease severity scores and lifetime lyso-Gb3 exposure at diagnosis

**Figure 1:**
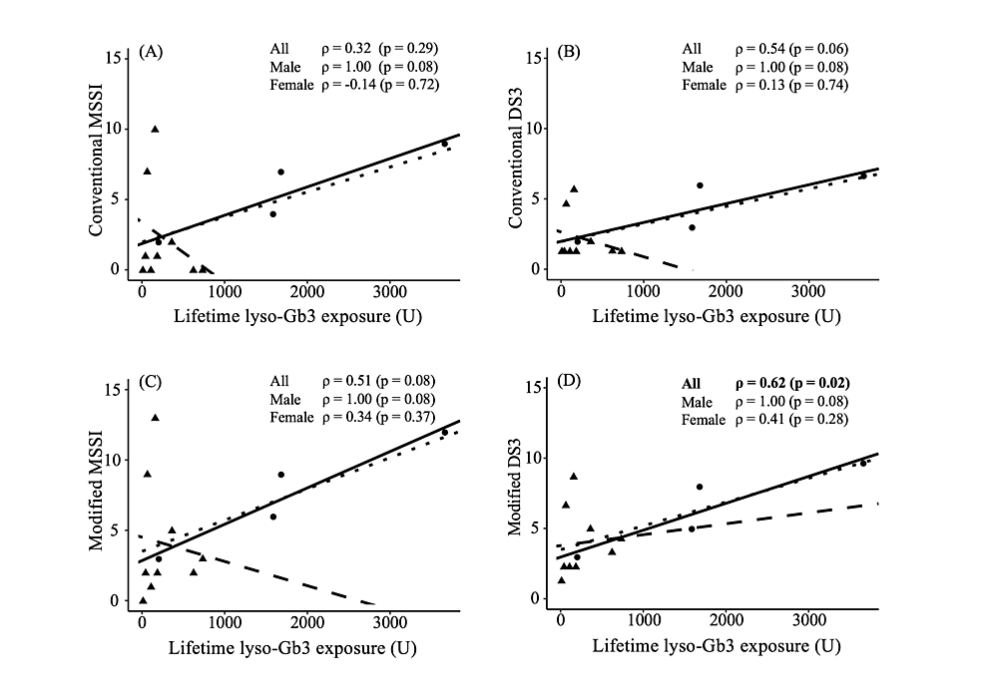
Correlations between lifetime lyso-Gb3 exposure and severity scores. The correlations of lifetime lyso-Gb3 exposure with (A) conventional MSSI score, (B) conventional DS3 score, (C) modified MSSI score, and (D) modified DS3 score. Triangles and dashed lines represent females. Circles and solid lines represent males. The dotted lines represent all subjects combined. Correlations were evaluated using Spearman's correlation coefficients. Abbreviations: DS3, Disease Severity Scoring System; MSSI, Mainz Severity Score Index; lyso-Gb3, globotriaosylsphingosine.

We examined the correlation between conventional DS3 or MSSI scores and lifetime lyso-Gb3 exposure at diagnosis in all 13 patients. There was no significant correlation between conventional scores and lifetime lyso-Gb3 exposure (Figures 1A, 1B).

We also examined the correlations of the modified scores with lifetime lyso-Gb3 exposure. The modified DS3 score was significantly positively correlated with lifetime lyso-Gb3 exposure in all patients (P = 0.02, Figure 1D). Only in modified DS3, females also showed a non-significant positive correlation trend.

Longitudinal changes in plasma lyso-Gb3 during treatment

**Figure 2:**
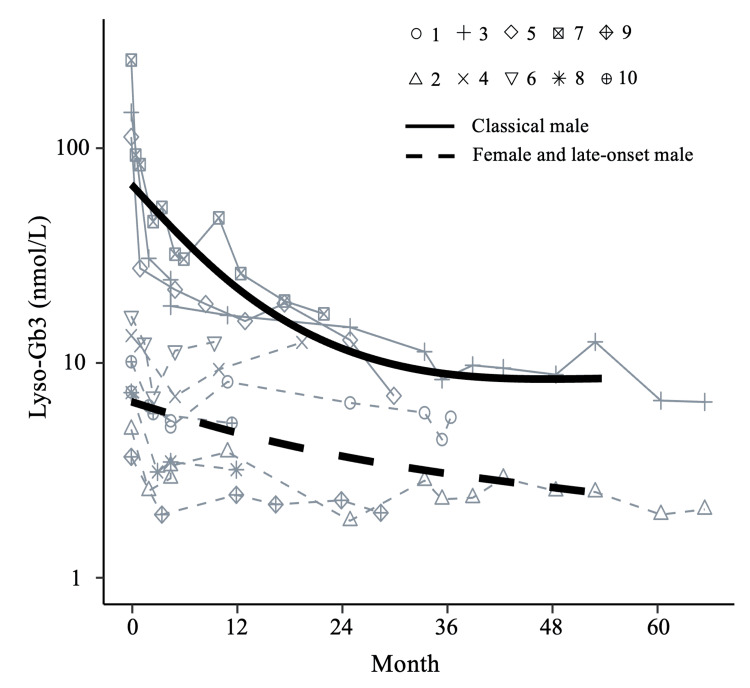
Longitudinal changes of lyso-Gb3 concentrations in Fabry disease patients after treatment initiation. Plasma lyso-Gb3 concentrations from the time of treatment initiation to the latest follow-up. Solid lines represent the changes in males with classical FD and dashed lines represent the changes in females with classical or late-onset FD and males with late-onset FD. Abbreviations: lyso-Gb3, globotriaosylsphingosine; FD, Fabry disease.

Longitudinal changes in plasma lyso-Gb3 concentration during treatment were examined in 10 of the 13 patients (patients 1-10; Figure 2). Before the start of treatment (day 0), males with classical FD (patients 3, 5, and 7) had particularly high baseline plasma lyso-Gb3 concentrations that decreased rapidly within a year of treatment initiation. In the female patients and late-onset male patients, there was a moderate and slow decrease in the plasma lyso-Gb3 concentration after treatment initiation.

Longitudinal changes in MSSI and DS3 scores during treatment

**Figure 3:**
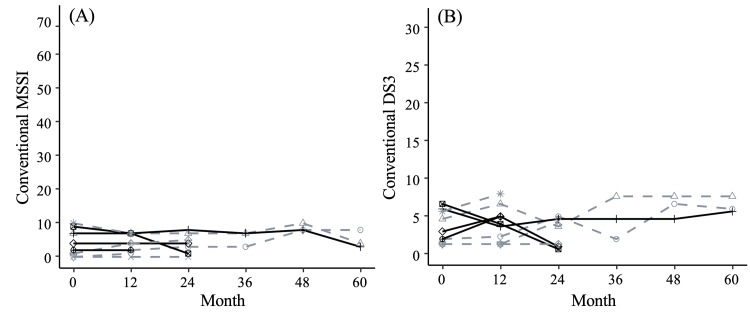
Longitudinal changes of conventional scores in Fabry disease patients after treatment initiation. (A) Conventional MSSI scores and (B) conventional DS3 scores. Solid lines represent changes in males and dashed lines represent changes in females. Abbreviations: DS3, Disease Severity Scoring System; MSSI, Mainz Severity Score Index.

Longitudinal changes in the conventional MSSI and DS3 scores during treatment were also examined in the 10 patients with follow-up data (Figure 3). In all patients, the scores remained nearly stable throughout the follow-up period. Changes in the severity score were likely due to fluctuations in the eGFR value and pain attacks. The eGFR values fluctuated close to normal, and patients’ pain seemed worse when assessed in the summer. These items therefore did not persistently worsen (data not shown). All patients were also assessed as stable by using FASTEX.

## Discussion

Our investigation in early-diagnosed patients revealed that the modified DS3, with additional items for early diagnosis, namely, uMB numbers, phenotype of classical form, and history of pain attacks, was positively correlated with lifetime lyso-Gb3 exposure at diagnosis.

Subjects could be diagnosed in the early phase of the disease, in the absence of advanced organ involvement, by using not only general screening tools such as echocardiography, blood tests, urine tests, GAL activity, and genetic testing but also additional tools such as uMBs and picking up at-risk relatives from family history.

The MSSI was originally developed for use in males with classical FD, whereas the DS3 was developed for easy use in the general FD patient population [18]. The DS3 has a patient-reported domain as well as clinical domains scored by laboratory data. In females with either form and males with the late-onset form, the DS3 may be more sensitive than the MSSI [19]. The FASTEX was developed to assess the clinical stability or disease progression between two consecutive evaluations. It has been reported to be more sensitive for assessing stability or disease progression than the MSSI and the DS3 [30]. However, these scores were of low value and insensitive in our patients without cardiac or renal impairment because the conventional scores consist mainly of organ manifestation items. A useful method of evaluating disease severity in children, adult females, and asymptomatic patients is needed [31,32].

In FD, early diagnosis, ideally during childhood, is important [21,32]. Nevertheless, the variable nature of the symptoms can lead to a delay between symptom onset and diagnosis [33]. Particularly, FD in childhood can be mistaken for other diseases, such as rheumatological problems, because of the nonspecific signs of lethargy and myalgia [34]. In our experience, such children have been misdiagnosed with orthostatic dysregulation or psychological problems because of general fatigue, school refusal, or nonspecific gastrointestinal symptoms. Additionally, adolescents or young adults may not complain of transient pain or hypohidrosis because pain improves over time [35], and they may have had those symptoms from early childhood and not consider them clinically meaningful symptoms. For early-diagnosed patients, episodes of past pain are important information. However, past pain cannot be assessed because conventional scores evaluate active pain. To facilitate diagnosis in children, a simple examination or screening test is needed. Potentially useful approaches are interviewing about family history, screening by enzyme assay using a dried blood spot [36,37], or checking for uMBs. uMBs, which are observed in the urine by light microscopy as laminated bodies with a whorled shape, are specific findings in FD. uMBs originate mainly from podocytes that have accumulated GL3 and are detected before proteinuria develops [38-40]. In a previous report, a high renal pathological score was observed in female patients with only uMBs without albuminuria [41]. This suggests that uMBs are potential early biomarkers of renal injury. Furthermore, a recent study showed that the degree of uMB excretion correlated with podocyte vacuolation and ERT reduces uMB excretion [40,42]. Podocyturia has also been reported as an early marker of renal injury, but detection requires staining [43]. Although the detectability of uMBs depends on the skill and experience of the technician, it can be a convenient indicator because of only using light microscopy. uMBs are not suitable on their own for assessing severity but they could be useful for assessing severity in children and asymptomatic patients if their scores were added to a conventional scoring system. Other early markers of cardiac involvement include troponin T and cardiac MRI (including T1 mapping) [44,45]. These were excluded from our study because they were not measured at baseline and their impact on severity and prognosis is unclear. In addition, a recent study showed that they should be managed according to phenotype because the clinical course of classical type and late-onset type is different [46]. Thus, we developed the modified scores, which have given a point for the phenotype of classical form and early markers such as uMBs and history of past pain attacks to the conventional scores. In this study, we determined phenotype based on genotype or family history as well as clinical features because early-diagnosed patients might not have enough symptoms to determine the phenotype, and we added points to the phenotype of classical form.

In FD males, plasma GL3 is high, whereas in symptomatic FD females, plasma GL3 is generally within the normal range [17,47,48]. In contrast, plasma lyso-Gb3 levels are high not only in males but also in females [17]. These findings suggest that lyso-Gb3 is more useful than GL3 for diagnosis. Lyso-Gb3 has recently been reported to be a pathogenic derivative of GL3 that increases extracellular matrix synthesis in podocytes [49]. We speculate that the process may be related to the progression of organ damage. Histological examination to elucidate the pathogenesis of lysosomal storage diseases, including FD, has revealed the direct involvement of GL3 accumulation and defective autophagy [50], as well as of microinflammation caused by cytokine production induced by lyso-Gb3 [49,51]. Therefore, prolonged exposure to lyso-Gb3 might have serious effects on organs. A previous study showed that lifetime lyso-Gb3 exposure was correlated with disease manifestations in male patients: mildly or moderately affected males had exposure levels between 1000 and 5000 U and severely affected male patients had exposure levels >5000 U [17], but few studies of lifetime lyso-Gb3 exposure have focused on females or children. Another report has shown that, in untreated women, disease severity scores increase with age [7], so the above hypothesis might be true for females as well.

To examine this association further, we assessed plasma lyso-Gb3 levels and calculated lifetime lyso-Gb3 exposure values at diagnosis for each patient according to previous reports [15,17,52]. In our patients, lifetime lyso-Gb3 exposures were less than 5000 U, and the severity score was mild, irrespective of gender or disease subtype. Lyso-Gb3 value might not be simply compared to previous reports because the value varies from laboratory to laboratory, and our results were consistent with a previous report [17]. The fact that our patients were relatively young and had received an early diagnosis may explain the low values. They had low exposure to lyso-Gb3 and possibly for this reason had not yet shown signs of progressive or symptomatic renal failure, symptomatic cardiac failure (hypertrophic myopathy), or cerebral infarction. One reason for these early diagnoses could have been that these patients were examined by physicians who were aware of FD and its clinical manifestations.

The modified DS3 was significantly correlated with lifetime lyso-Gb3 exposure in all patients taken together. Additionally, it was noteworthy that the female group showed a non-significant positive correlation trend only in modified DS3. Therefore, modified DS3 might be useful for assessment in early-diagnosed FD patients. However, in females, no significant correlations were found, even with the modified DS3, despite a trend toward correlation in the case of males. This may have been due to the insufficiency of the sample size for stratification by gender, as well as the heterogeneity of symptoms in females. Generally, FD is milder in female patients than in male patients [7,8]. At the end of the 1990s, we reported that a female patient with FD showed symptoms that were limited to the kidney because she had lyonization of the X chromosome that included a mutated GLA. At that time, it had not been confirmed that FD could be manifested in heterozygous females and that these individuals were not simply carriers of the pathogenic gene. Subsequent studies have shown that females have a wide spectrum of symptoms ranging from asymptomatic to severe, just as in males [9,10]. Previous studies have suggested that the skewing of X-chromosome inactivation is part of the reason why females have a wide spectrum of symptoms [53]. However, it has not been determined conclusively whether the degree and distribution of skewing of the organs in which X-chromosome inactivation is present are related to disease severity [31,53,54].

Finally, we estimated the longitudinal changes in plasma lyso-Gb3 levels and disease severity scores. Males showed more rapid decreases in plasma lyso-Gb3 concentration after the treatment initiation than did females. The cut-off value of lyso-Gb3 was 0.9 nmol/L (95th percentile of healthy individuals) in a previous study [55]. Note that none of the patients had a plasma lyso-Gb3 concentration lower than the cut-off value at any time during follow-up. However, the reduction in lyso-Gb3 concentration in response to ERT also decreases lyso-Gb3 exposure post-ERT initiation. Indeed, notably, the conventional disease severity scores of our subjects were low and stable, and although uMBs were persistently observed, their presence did not lead to proteinuria. This suggests that decreased exposure to lysoGb3 after ERT initiation may slow the progression of signs and symptoms and improve prognosis. We believe that this possibility should be evaluated in a future study by renal biopsy.

Our study was limited because of the insufficient sample size, follow-up periods, and the number of pedigrees. Moreover, there was a lack of weighting of additional items in the modified disease severity scores. Studies that include a large sample size of early-diagnosed FD patients and a long-term follow-up period are needed to validate our findings and to determine the effect of lifetime lyso-Gb3 exposure on long-term prognosis.

## Conclusions

Our study suggests that the modified DS3 score and lifetime lyso-Gb3 exposure are useful for diagnosing early FD and assessing the condition of FD patients.

Raising awareness of FD among primary care physicians will be essential to increase the rate of early diagnosis in the pediatric population. FD specialists should use lifetime lyso-Gb3 exposure and the modified DS3 at routine outpatient examinations to ensure that patients are motivated to continue lifelong treatment. Patients and their caregivers should be provided with an understanding of the natural history of the disease through discussions with FD specialists, medical geneticists, genomics counselors, and genomics nurses. In this context, the availability of non-invasive biomarkers of disease progression will allow patients and their caregivers to easily understand any changes in clinical status during treatment.
